# Letter to the editor regarding ‘A systemic review and meta-analysis on the efficacy and safety of ferumoxytol for anemia in chronic kidney disease patients’

**DOI:** 10.1080/0886022X.2022.2153065

**Published:** 2023-01-13

**Authors:** Linqiang Li, Huiqin Feng

**Affiliations:** Department of Internal Medicine, Tongxiang Chinese Medicine Hospital, Tongxiang, China

Dear Editor,

We took great interest in reading Zuo et al.’s article [1], in which the authors assessed the efficacy of ferumoxytol by conducting a meta-analysis on the current literature for chronic kidney disease (CKD).

They extracted 7 random controlled trials (RCTs) between January 1950 to October 2020 from 6 academic databases and concluded that ferumoxytol elevated hemoglobin levels, transferrin saturation, and ferritin levels in patients with CKD, compared to conventional iron supplement formulations. After examining the data distribution and analyzing hemoglobin, ferritin levels, and adverse event incidence between study groups receiving ferumoxytol and iron supplement formulations, the authors exacted data from 3315 CKD patients, from whom 2034 patients got ferumoxytol and 1281 people got placebo or supplements.

Next, the authors concluded that there was a small negative effect (Hedge’s *g* statistic: 0.24, Standard Error: 0.14, Variance: 0.02) when evaluating treatment-emergent adverse events (TEAEs). They also saw a medium positive effect in hemoglobin level (Hedge’s *g* statistic: 0.51, Standard Error: 0.09, Variance: 0.009) and transferrin saturation (Hedge’s *g* statistic: 0.39, Standard Error: 0.17, Variance: 0.02). Moreover, a large positive effect (Hedge’s *g* statistic: 0.88, Standard Error: 0.36, Variance: 0.13) could be detected in the ferritin level. However, we had some confusion that needed to be clarified after reading this article.

First, in the ‘data search strategy’ section, the authors claimed that either randomized controlled trials, quasi-randomized controlled trials, controlled clinical trials, prospective observational trials with control groups, or retrospective trials would be included. However, only 7 RCTs were selected in the end, which did not conform to their search strategy. We wonder why they excluded all other non-RCTs rather than following the rule they made in the data search strategy section. Even if those non-RCTs could not be comparable to RCTs, they could be a useful supplement. Otherwise, they should better revise the including criteria by removing all other non-RCTs, if they just wanted to search for RCTs.

Second, some patients were recruited twice in this meta-analysis. In Lu et al.’s study [2], there were 3 trials conducted with some data that had been published ahead. According to Lu’s quotation, some of the published data had been reported in Singh et al.’s article [3], which was also extracted into this meta-analysis.

To be precise, Lu’s research was an FDA report consisting of two randomized, open-label, controlled clinical trials evaluating participants with non-dialysis dependent CKD and a third one recruiting patients with CKD stage 5D undergoing hemodialysis. Meanwhile, Singh and his/her partners claimed that their study was a phase 3, randomized, double-blind, placebo-controlled, crossover, multicenter study of the safety profile of ferumoxytol compared with normal saline, conducted at 47 US sites, and their patients were with CKD stages 1 to 5 and 5 D compared, which was in accord with Lu’s study. In consequence, there must be repeated patients in these two studies and this duplicative data could erode the accuracy and reliability of the conclusion.

Another similar problem could be found in another 2 articles written by Strauss et al. [4] and Macdougall et al. [5]. In these two studies, two clinical trials comparing the efficacy and safety of ferumoxytol with iron sucrose for the treatment of Iron deficiency anemia (IDA) were analyzed and one of them shared the same clinical trial with Macdougall’s (ClinicalTrials.gov identifier: NCT01052779). In Strauss’s study, the authors performed their study using 2 completed clinical trials. In the first trial, researchers assessed the safety and efficacy of ferumoxytol with iron sucrose for the treatment of IDA in CKD adult patients with or without dialysis. In the second one, patients with any unexplained IDA and a history of unsatisfactory or inability to receive oral iron therapy were selected. The first trial was registered with an identifier of NCT01052779. Meanwhile, in Macdougall’s research, both hemodialysis and non-dialysis patients assigned to ferumoxytol received two IV injections of 510 mg (17 mL; no faster than over 17 s) within 563 days for an accumulative dose of 1.02 g. This randomized, open-label, multicenter, international, phase 2 trial shared the same clinical trial number as Strauss’s study, also known as NCT01052779. Consequently, these data were analyzed repeatedly in this meta-analysis.

Third, all the recruited patients were diagnosed with CKD stages 1–5 but some trials only included CKD patients with stage 5D. This difference could greatly increase the heterogeneity of this research and decline the reliability of the conclusion. We could not find any heterogeneity test about this yet.

Fourth, publication bias was assessed by funnel plot although there were less than 10 included studies. Generally, a funnel plot was not compatible as symmetries were difficult to detect and Harbord Test could be applied on this occasion.

Finally, we are grateful for the authors’ work and their insightful conclusions.
